# High TRB3 expression induces chondrocyte autophagy and senescence in osteoarthritis cartilage

**DOI:** 10.18632/aging.204066

**Published:** 2022-07-01

**Authors:** Yanqing Gu, Ren Yan, Yang Wang, Yiwen Zeng, Qingqiang Yao

**Affiliations:** 1 Department of Orthopedics, Nanjing First Hospital, Nanjing Medical University, Nanjing, Jiangsu, China; 2 Cartilage Regeneration Center, Nanjing First Hospital, Nanjing Medical University, Nanjing, Jiangsu, China; 3 Digital Medicine Institute, Nanjing Medical University, Nanjing, Jiangsu, China

**Keywords:** TRB3, autophagy, senescent cell, osteoarthritis

## Abstract

Objective: Osteoarthritis is closely related to aging. Tribbles homologue 3 (TRB3) is found to display age-related expression and contributes to the regulation of cell proliferation, differentiation and fibrosis. In this study, we aimed to investigate the potential involvement of TRB3 in cartilage autophagy and aging in osteoarthritis.

Methods: Cartilage tissue samples were collected from osteoarthritis patients who received joint replacement and cadaveric donors. In osteoarthritis cartilage tissue, we analyzed autophagy- and senescence-associated proteins using immunohistochemistry and western blot (WB), *in vitro*, to confirm the role played by TRB3 in the process of autophagy, cell senescence, and inflammation, small interfering RNA (siRNA) was used for TRB3 knockdown in cells.

Results: We found increased level of p62, decreased level of microtubule-associated protein 1A/1B-light chain 3 (LC3) and beclin-1 in cartilage, and increased level of p16 and p21 in tissue samples collected from osteoarthritis patients, indicating decreased autophagy and increased cell senescence. TRB3 knockdown significantly rescued, *in vitro*, the reduced autophagy and elevated cell senescence in human chondrocyte.

Conclusions: Interfering with TRB3 expression in cartilage may serve as a target in the prevention and treatment of age-related osteoarthritis.

## INTRODUCTION

Osteoarthritis is the most common type of arthritis [1]. Clinical treatment of osteoarthritis is limited due to the lack of effective therapeutic methods, and joint replacement surgery is the most commonly applied treatment. The incidence of osteoarthritis is significantly increased with age due to aging-associated cartilage tissue degradation.

Generally, chondrocytes play an important role in maintaining the homeostasis of cartilage. However, aging-associated cellular alterations, such as cell senescence [2], oxidative stress [3], inflammation [4], autophagy defects [5], and mitochondrial dysfunction [6], result in the functional decline of chondrocytes. These alterations collectively affect cartilage integrity and enhance osteoarthritis development. However, the underlying molecular mechanism of this process is still unclear.

Tribbles homolog 3 (TRB3) displays age-related expression patterns [7]. TRB3 also plays an important role in regulating cell proliferation, survival, and differentiation. TRB3 is expressed in human chondrocytes. Previous studies have indicated increased TRB3 levels in cartilage and isolated chondrocytes of osteoarthritis patients. TRB3 levels in cartilage and chondrocytes also increased due to endoplasmic reticulum stress in osteoarthritis. TRB3 is an inhibitor of Akt activation. Increased TRB3 production contributes to increased cell death and reduced response to insulin-like growth factor-I as observed in osteoarthritis cartilage [8].

TRB3 contributes to the regulation of autophagy through its interaction with autophagic receptor p62 and inhibits p62 binding with microtubule-associated protein 1-light chain 3 (LC3) and ubiquitinated substrates. This process subsequently leads to the accumulation of p62 protein and suppression of autophagic/proteasomal degradation. A previous study has demonstrated the potent antitumor effects of TRB3/p62 interaction interruption [9].

Robust evidence suggests the correlations among autophagy deficiency, loss of cellular homeostasis, cartilage aging, and osteoarthritis development [10, 11]. Moreover, cell senescence and autophagy are essential for maintaining cellular homeostasis and can be targeted in treating age-associated diseases [12]. Recent studies have suggested reduced cartilage autophagy in osteoarthritis [13] and the protective effect of autophagy in preventing chondrocyte degradation [14].

This study aimed to investigate further the function of TRB3 in osteoarthritis development. Results showed reduced beclin1 and LC-3II levels in the cartilage of osteoarthritis patients compared to healthy controls (HCs). TRB3 knockdown significantly rescued decreased autophagy and reduced cell senescence in chondrocytes *in vitro*. These findings suggested the important regulative role played by TRB3 in tumor necrosis factor-α (TNF-α)-induced autophagy in human chondrocytes. This study provided the first evidence demonstrating the correlations among TRB3 levels, aging, and autophagy in human chondrocytes and its critical role in osteoarthritis development.

## RESULTS

### Severe cartilage degradation, altered TRB3, and autophagy-related gene expression in osteoarthritis patients

**Figure 1:**
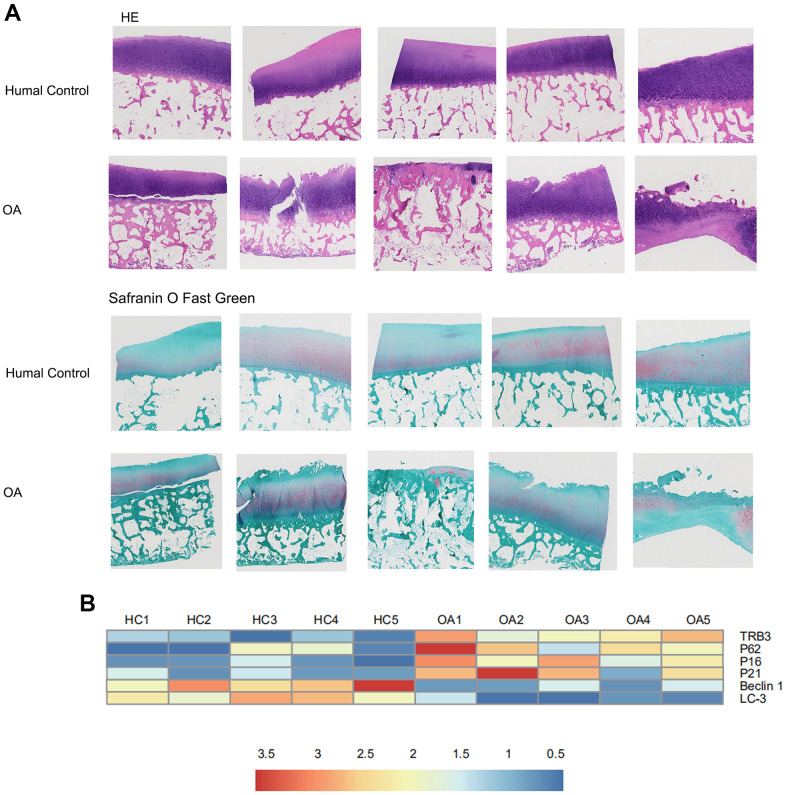
**The results of histology for cartilage degradation in the control and osteoarthritis groups.** (**A**) Representative photomicrographs HE staining and Safranin-O/Light Green Red staining of joint pathological sections of normal joints and OA model, (**B**) Heatmap analyses of the key molecules regulated in OA patients and healthy controls based on transcriptome analysis. Green, low expression levels; red, high expression levels.

**Figure 2:**
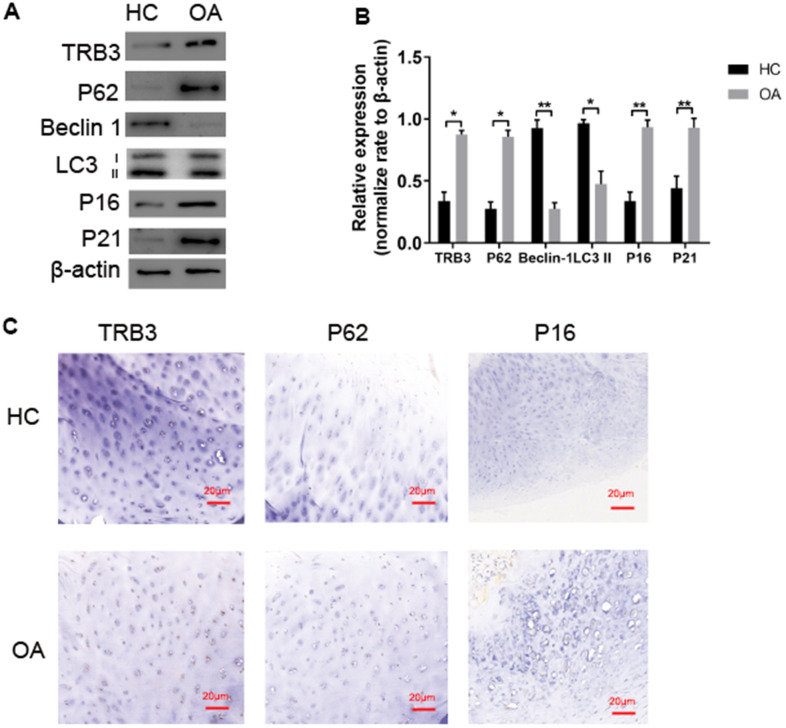
**TRB3 is increased in human and knee articular cartilage.** (**A**, **B**) Blots were stripped and reprobed with β-actin as a loading control. Densitometric analysis for protein levels of TRB3, P62, P16, P21, Beclin and LC3B. (**C**) Articular cartilage sections of HC or OA were analyzed immunohistochemically for TRB3, P62, and P16. The results are described as the mean ± SD. *P < 0.05, **P < 0.01 vs. normal group.

In Figure 1A, according to histological and immunohistochemical results, cartilage degradation in osteoarthritis patients was significantly more severe than in HCs, as indicated by the deeper extended cartilage surface destruction and proteoglycan disorder. RNA-seq analysis was conducted between samples collected from osteoarthritis patients and HCs. The results were displayed in a heat map (Figure 1B). In osteoarthritis patients, TRB3, p62, p16, and p21 mRNA expression was upregulated, whereas LC3-II and beclin1 mRNA expression was upregulated (Figure 2B).

### TRB3 protein levels, autophagy markers, and senescent markers in the cartilage of osteoarthritis patients and HCs

WB analysis and immunohistochemistry of human chondrocytes isolated from cartilage tissue collected from osteoarthritis patients and HCs were carried out. TRB3 protein levels were significantly increased in articular cartilage (Figure 2A, 2C). The autophagy-related protein p62 level was significantly increased in osteoarthritis patients, indicating p62 accumulation. LC3-II and beclin1 protein levels significantly decreased in osteoarthritis patients compared to HCs. p16 and p21 protein levels significantly increased in osteoarthritis patients compared to HCs (Figure 2A). Consistent results of p62 and p16 levels were also revealed by immunohistochemistry results (Figure 2C).

### Increased TRB3 expression in TNF-α-induced osteoarthritis model *in vitro*

**Figure 3:**
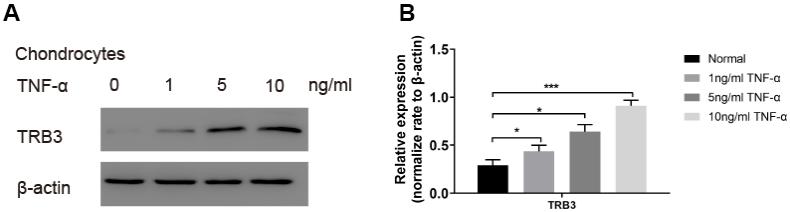
**The TRB3 expression in TNF-α-triggered chondrocytes.** Chondrocytes were treated with 0, 1, 5, 10 ng/ml TNF-α for 4 days. (**A**, **B**) The TRB3 expression was assessed via blots, and normalization to the levels of β-actin. The results are described as the mean ± SD. *P < 0.05, **P < 0.01 vs. normal group.

For further investigation of the potential role played by TRB3 in chondrocytes in osteoarthritis patients, the TRB3 level was subsequently examined in a TNF-α-induced osteoarthritis model *in vitro*. TNF-α treatment dose-dependently increased the TRB3 protein level in chondrocytes (Figure 3). This finding suggested that the TRB3 level was upregulated in response to stimulating inflammatory factors in chondrocytes.

### TRB3-dependent autophagy in chondrocytes

**Figure 4:**
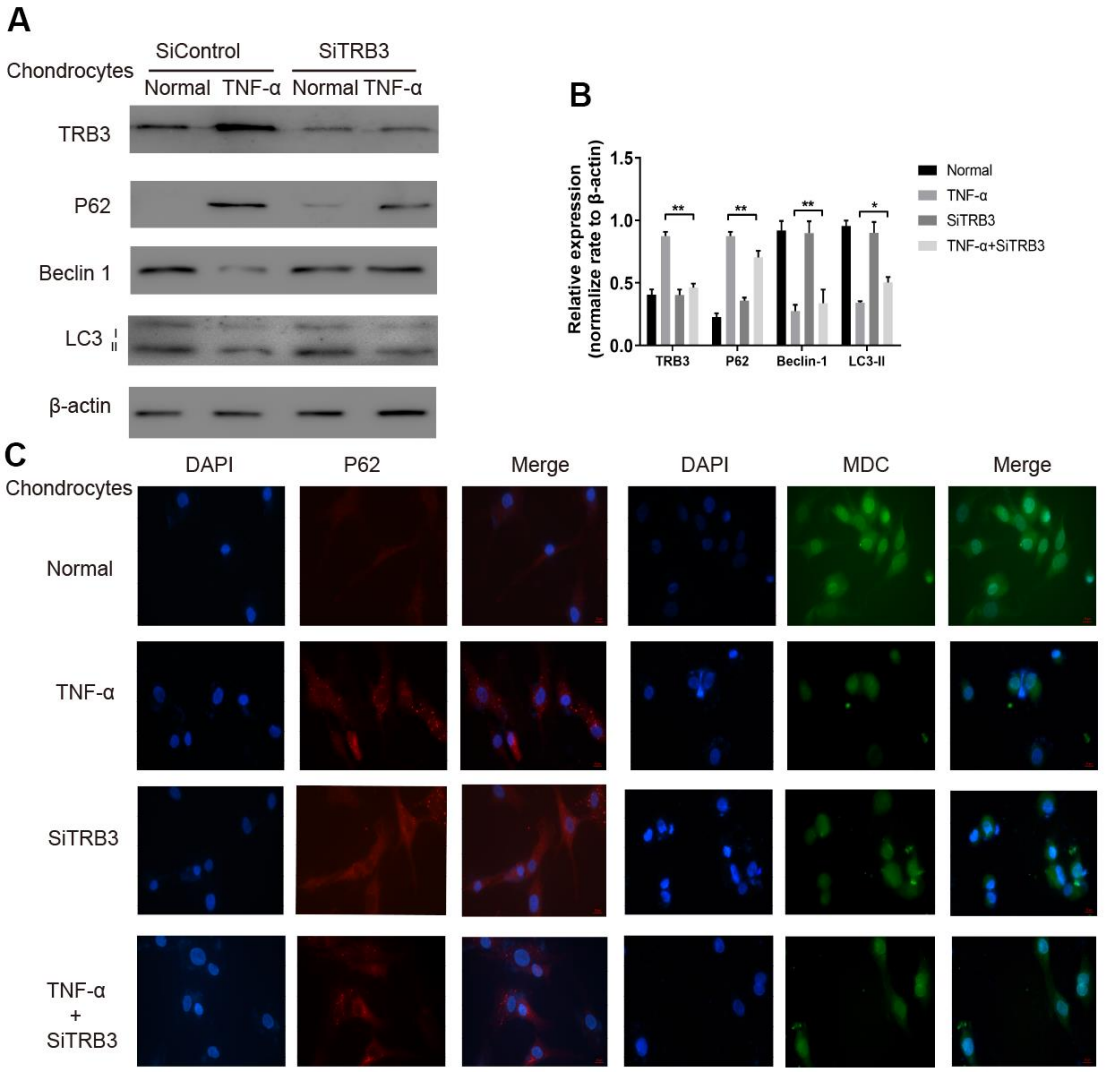
**Chondrocyte autophagy is dependent on TRB3.** (**A**, **B**) Protein expression of TRB3, P62, Beclin 1 and LC3 in isolated chondrocytes from young healthy treated with TNF-α, TRB3 siRNA or control siRNA. (**C**) Chondrocyte autophagic activity was detected by Immunofluorescence of P62 and MDC staining. The results are described as the mean ± SD. *P < 0.05, **P < 0.01 vs. normal group.

The potential effects of TRB3 knockdown on autophagy were subsequently investigated in chondrocytes. Using WB, TRB3 knockdown significantly downregulated p62and upregulated LC3-II and beclin1 levels (Figure 4A, 4B). Consistent results of the p62 protein level were obtained by immunofluorescence (Figure 4C). These results indicated that autophagy was inhibited by TNF-α stimulation. The p62 protein level was decreased by TRB3 knockdown in chondrocytes. Moreover, MDC staining revealed that autophagy was promoted by TRB3 knockdown in chondrocytes stimulated with TNF-α (Figure 4C).

### TRB3-dependent senescence in chondrocytes

**Figure 5:**
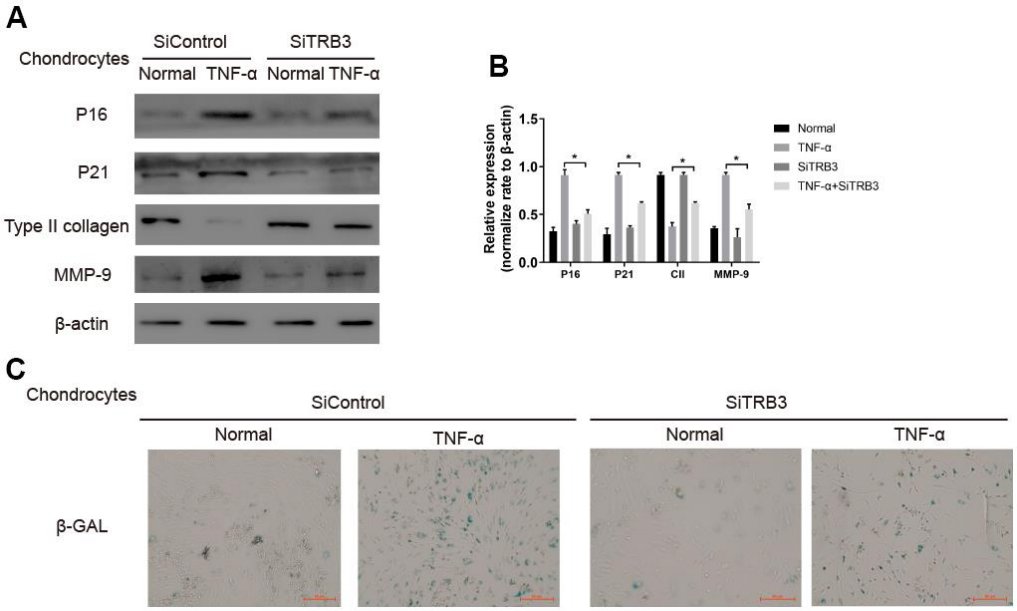
**Chondrocyte senescent is dependent on TRB3.** (**A**, **B**) Western blotting analyses of senescent markers p21, p16, type II collage and MMP-9 with β-actin as a loading control. (**C**) Representative graphs of cells stained for β-gal activity. The results are described as the mean ± SD. *P < 0.05, **P < 0.01 vs. normal group.

TRB3 knockdown inhibited the process of cell senescence in chondrocytes, as revealed by significantly decreased p16 and p21 levels (Figure 5A, 5B). The number of SA-β-Gal-positive cells in TNF-α-stimulated chondrocytes significantly increased compared to HCs. TRB3 knockdown partially rescued the increased SA-β-Gal-positive cell number in TNF-α-stimulated chondrocytes (Figure 5C).

## DISCUSSION

This study demonstrated a significantly increased TRB3 level in osteoarthritis patients. TRB3 was a pseudokinase, and its gene expression was induced under stress events in cells. TRB3 was also demonstrated to play a critical role in regulating cell survival [15–18].

Previous research demonstrated that TRB3 interacted with autophagy protein p62 and interrupted its binding with ubiquitin-labeled substrates and LC3. TRB3-p62 interaction subsequently led to the accumulation of p62 protein and suppression of autophagy [9, 17], indicating the important association between TRB3 levels and cellular autophagy activities. For the first time, this study investigated the role of TRB3 in regulating autophagy in chondrocytes and its potential function in osteoarthritis development.

Abundant evidence indicated the association between autophagy deficiency and the process of aging in mouse and human knee articular cartilage tissue. This phenomenon was directly revealed by the reduction of key regulators of autophagy, such as Unc-51-like autophagy activating kinase 1, beclin1, and LC3 [10]. A previous study demonstrated 20 downregulated autophagy-associated genes in osteoarthritis patients, including LC3 and beclin1, compared to HCs [10]. Consistently, LC3 and beclin1 mRNA and protein levels were downregulated in the osteoarthritis group.

Autophagy was demonstrated to produce protective effects during osteoarthritis development by preventing apoptosis and aging of chondrocytes. Results showed that TRB3 knockdown in chondrocytes caused p62 reduction and LC3-II and beclin1 elevation (Figure 4). The interaction between TRB3 and p62 directly caused p62 accumulation and thus inhibited p62-mediated autophagy. Results also showed that an increased TRB3 level suppressed autophagy activities. The difference in LC3B levels in the presence and absence of chloroquine is more under autophagy-induced conditions, indicating that autophagic flux is increased. This study found that chloroquine blocked TRB3-siRNA-induced autophagy in chondrocytes (Supplementary Figure 1).

Previous studies found that the activation of autophagy produced protective effects by preventing chondrocyte cell death, cartilage senescence, and osteoarthritis development [10, 11]. Articular cartilage tissue collected from osteoarthritis patients displayed features of senescence (as indicated by increased p16 levels), DNA lesions, and SA-β-Gal activation [19–21]. The potential effects of TRB3 knockdown on cartilage aging were further investigated. TRB3 knockdown caused the reduction of p16 and p21 levels. TRB3 knockdown also inhibited SA-β-Gal activities in TNF-α-stimulated chondrocytes. These findings suggested the inhibitory effects of TRB3 knockdown on cell senescence.

**Figure 6:**
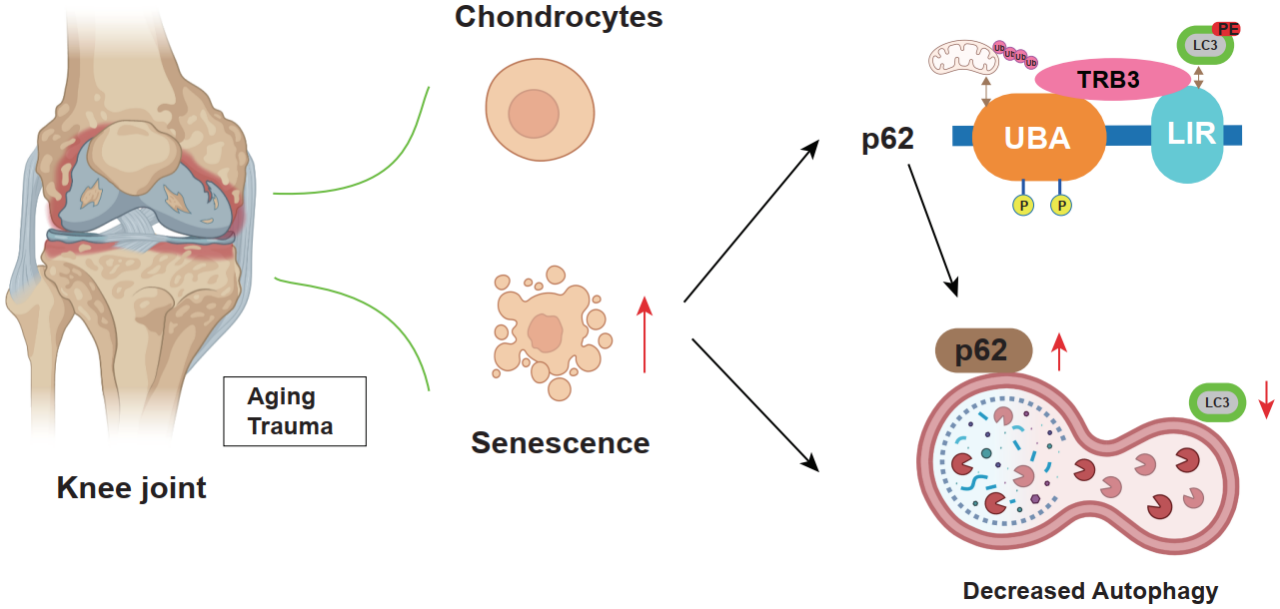
Schematic diagram illustrates the role of TRB3 in steoarthritis development and progression.

Collectively, results suggested the promoting effects of TRB3 in osteoarthritis development via the inhibition of autophagy and activation of the p62 signaling pathway (Figures 3, 4, 6). TRB3 knockdown alleviated osteoarthritis through the enhancement of autophagy and reduced senescence. These findings provided robust evidence suggesting that TRB3 could be potentially targeted in the prevention and clinical treatment of age-associated osteoarthritis.

## MATERIALS AND METHODS

### Reagents

TNF-α (R&D Systems) and TRB3-small interfering RNA (siRNA; Sigma-Aldrich) were commercially purchased. TNF-α and TRB3-siRNA were freshly prepared before use. Antibodies against TRB3 (Pro Sci; lot #8099) LC3-B (abcam; lot #ab192890), beclin1 (abcam; lot #ab210498), p16 (abcam; lot #ab189034), p21 (abcam; lot #ab107099), and p62 (abcam; lot #ab109012), matrix metalloproteinase-9 (abcam; lot #ab76003), and type II collagen (Santa Cruz Biotechnology; lot #sc-52658) were used in this study.

### Human cartilage collection and chondrocyte isolation

Cartilage tissue samples of osteoarthritis patients (71–89 years old) were collected during joint replacement operations. Osteoarthritis patients with severe limitations in joint movement and severe joint pain in the knee above K-L grade III level met the criteria for surgery and were suggested to receive total hip arthroplasty. Cartilage tissue of HCs was collected from cadaveric donors without the bone disease (70–85 years old) from the Anatomy Teaching and Research Office of Nanjing Medicine University (control group, n = 5; 3 females and 2 males; mean age, 75 ± 3.3 years). Tissue samples were collected with informed donor consent in full compliance with national and institutional ethical requirements and the Declaration of Helsinki.

For chondrocyte isolation, cartilage tissue collected in the previous section was cut into pieces and incubated overnight in Dulbecco’s modified Eagle medium containing 1 mg/mL collagenase A (Roche Pharmaceuticals) at 37° C. Isolated chondrocytes were subcultured once (passage 2) and ready for use.

### RNA-sequencing analysis

Total RNA was extracted from osteoarthritis patients or HCs. The extracted RNA was quantified using NanoDrop ND-1000 (NanoDrop, Wilmington, DE, USA). RNA integrity was assessed by Bioanalyzer 2100 (Agilent, Santa Clara, CA, USA) with an RNA integrity number of >7.0 and confirmed by electrophoresis with denaturing agarose gel. Then, 2 × 150 bp paired-end sequencing (PE150) of each sample was performed on an Illumina Novaseq™ 6000 (LC-Bio Technology Co., Ltd., Hangzhou, China) according to the vendor’s recommended protocol for mRNA library construction and sequencing. RNA-seq analysis was carried out according to the previously reported protocol [22].

### Cell culture

The isolated chondrocytes from osteoarthritis patients and HCs were plated in six-well plates (2 × 105 cells/well) and serum-starved overnight. Chondrocytes were treated with TNF-α at final concentrations of 1, 5, and 10 ng/mL for 4 days in a serum-free medium at 37° C in a 5% CO2 incubator. Before TNF-α treatment, chondrocytes were pretreated with TRB3-siRNA (5 mM) for 48 h at 37° C. Chondrocytes treated with ShControl were used as control. Cells were harvested for Western blotting (WB).

### Hematoxylin-and-eosin staining

Cartilage tissue was fixed with 10% neutral buffered formalin (pH 7.4), decalcified in 20% EDTA solution, and embedded in paraffin. Tissue sections (5 mm) were prepared and stained with H&E, toluidine blue, and safranin O-Fast Green.

### Immunohistochemical analysis

Sections of cartilage tissue were examined by immunohistochemistry. Antigen retrieval was carried out in citrate buffer. Primary antibodies against TRB3, p62, and p16 were incubated with the sections overnight. Horseradish peroxidase-conjugated secondary antibodies were incubated with the sections for 1 h. Diaminobenzidine was used for the visualization of positive staining. Images were captured under an optical microscope (Leica Microsystems). The number of positively stained cells was evaluated using Image-Pro Plus (Media Cybernetics).

### Western blot

Protein samples were prepared in loading buffer, separated by sodium dodecyl sulfate-polyacrylamide gel electrophoresis, and transferred to a polyvinylidene fluoride membrane. WB was carried out according to the previously described protocol [23].

### Immunofluorescence staining

Chondrocytes were plated on glass plates coated in a six-well plate. Chondrocytes on the glass plates were fixed in 4% paraformaldehyde for 1 h, treated with 0.5% Triton X-100 for 10 min, and blocked in 1% bovine serum albumin solution. Primary antibodies against p62 and secondary antibodies were subsequently incubated with the glass plates. 2-(4-Amidinophenyl)-6-indolecarbamidine dihydrochloride solution was used to visualize the nucleus. Images were obtained by Leica microscopy.

### Monodansylcadaverine staining

Detection of autophagic vacuoles was carried out using monodansylcadaverine (MDC) staining. Generally, chondrocytes were plated in a six-well plate. Chondrocytes were subsequently collected and washed with phosphate-buffered saline. An aliquot of 80 μL cell suspension was incubated with 20 μL MDC staining solution (30 min) and plated on glass slides. Autophagic vacuoles were evaluated under a fluorescence microscope.

### β-galactosidase staining

The senescence β-gal staining kit was used for β-gal staining in chondrocytes, and X-Gal was used as a substrate for the reaction. Cell senescence was indicated by the increased level of β-gal activity. Positive cells were stained aquamarine green. Results were analyzed and displayed as the percentages of the SA-β-gal-positive cell counts to the total cell counts.

### Statistical analysis

All statistical analyses were carried out using GraphPad Prism version 7.00. Technical replicates (each donor) and biological replicates (all donors) were performed for statistical analysis. Repeated measurement of one-way analysis of variance with multiple comparison correction (Dunnett’s test) was used for the comparison between several groups, and a t-test was used for the comparison between two groups.

## Supplementary Material

Supplementary Figure 1
