# Supplementary material for: High TRB3 expression induces chondrocyte autophagy and senescence in osteoarthritis cartilage
Source: Aging (Albany NY). 2022 Jul 1;14(13):5366–75. doi: 10.18632/aging.204066 (PMC9320551; doi:10.18632/aging.204066)
Supplement: Supplementary Figure 1 [file aging-14-204066-s001.pdf]

SUPPLEMENTARY FIGURE

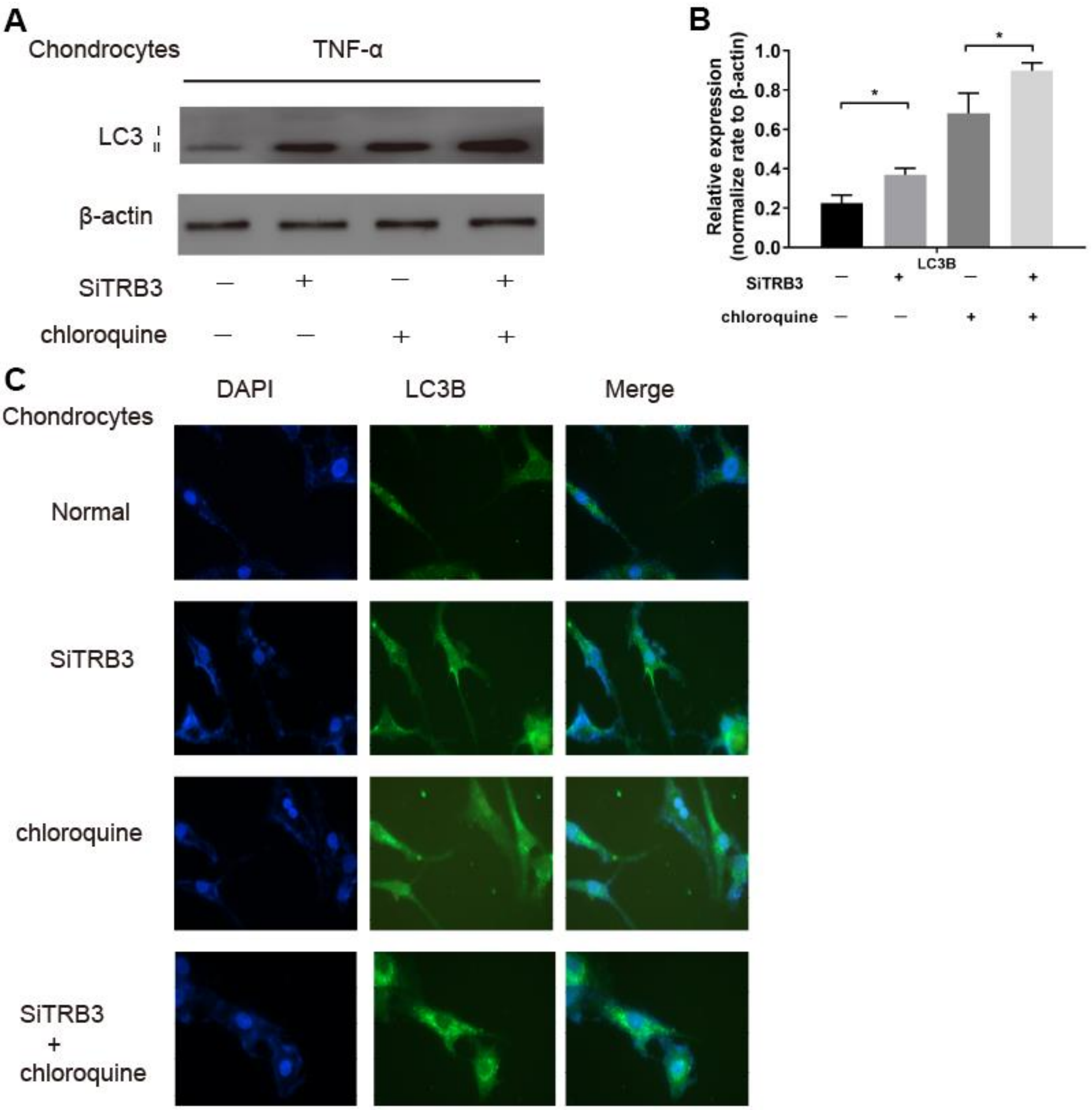

Supplementary Figure 1. Chloroquine block TRB3-siRNA induced autophagy in chondrocytes. (**A**, **B**) protein expression of LC3B in isolated chondrocytes from young healthy treated with TNF-α, SiTRB3 or chloroquine. (**C**) Chondrocyte autophagic activity was detected by Immunofluorescence of LC3B staining. The results are described as the mean ± SD. *P < 0.05, **P < 0.01 vs. normal group.
